# What Factors Will Influence Chinese International Traveling for Leisure in the Post-COVID-19 Era: Role of Health Priorities and Health-Related Information Literacy

**DOI:** 10.3390/healthcare11030315

**Published:** 2023-01-20

**Authors:** Saba Batool Wadhar, Riffat Shahani, Rongting Zhou, Ahmad Nabeel Siddiquei, Qing Ye, Fahad Asmi

**Affiliations:** 1 Department of Science Communication, University of Science and Technology of China, Hefei 230026, China; 2 Bond Business School, Bond University, Gold Coast, QLD 4226, Australia; 3 Department of Economic Management, College of Information Engineering, Fuyang Normal University, Fuyang 236037, China

**Keywords:** fear of new pandemic outbreak, pandemic seriousness, information literacy, avoiding international travel for leisure, digital tourism

## Abstract

China used to be the world’s leading nation in terms of international (outward) tourism till the COVID-19 outbreak. However, due to the COVID-19 crisis, several new macro and micro-level factors might affect their international (outward) traveling behavior. The purpose of the current research was to examine the avoidance of international traveling for leisure in the Chinese population. The goal of the study was to highlight the importance of information self-efficacy and digital literacy as the key factors influencing tourists’ traveling readiness. To achieve the goal, the study adapted the quantitative instruments from existing sources to map media exhaustion, information overload, and perceived health concerns, i.e., perceived effectiveness of health-protective measures, fear of new possible outbreaks, and pandemic crisis at source and destination. Chinese citizens’ opinions were collected during the third quarter of the year 2022. Specifically, the quantitative survey from China collected a total number of 1308 respondents. This study used the statistical analysis software SPSS to analyze collected data. The findings conclude that the role of media is pivotal to shaping and predicting future trends in tourism preferences, perception of protective measures against COVID-19, and perceived seriousness of the pandemic crisis in the Chinese population. In addition, technology readiness (as hard self-efficacy) and health-related information literacy (soft self-efficacy) are critical to cope with the dark aspects of information exhaustion, overload, and pandemic seriousness in the post-truth era. The study is unique, as it examines the role of the seriousness of the pandemic at its source and destination and fear of new outbreaks simultaneously, underlining the potential future of immersive tourism (i.e., virtual reality, augmented reality, or mixed reality-based tourism). This study has drawn interesting theoretical and practical implications for researchers, policymakers, and academicians.

## 1. Introduction

The World Health Organization (WHO) declared the novel Coronavirus (COVID-19) outbreak a pandemic crisis on 11 March 2020 [1]. It directly and indirectly affected public domestic and international travel movements around the globe [2]. The tourism and hospitality industry was one of the worst-hit economic sectors during the COVID-19 pandemic [3]. Statistically, the worldwide tourist economy was hit with a loss of up to 60–80% as compared to the year 2019 (UN News, 2020). Under the influence of the pandemic, tourists’ choices of destination are more inclined towards domestic destinations rather than foreign countries [4]. At the same time, to fulfill their tourism/leisure needs, People could desire exclusivity and pick well-known vacation sites that they believe to be safe for tourist activities, such as scenic spots with fewer tourists and less traffic. The development of information technology has aided the tourism revolution and given rise to a new kind of travel: virtual tourism. Virtual tourism creates new opportunities for changes in tourists’ behaviors and, subsequently, significant opportunities for tourism organizations to use technology to gain a competitive advantage [5].

In light of recent socio-economic dynamics in the national and international sphere and the post-truth era of information technology, politicization, international relations, and ICT-based communication are at a new height. For instance, there is a very different understanding of the COVID-19 crisis globally, as each of the responsible nations is handling the pandemic in its own best possible way [6]. The seriousness of these concerns varies due to the difference in first-hand experience and knowledge in society about the ongoing pandemic and its severity. The study contributes, as it addresses the role of the degree of media exhaustion [7], informational disbelief in the post-truth era [8], fear of new epidemic/pandemic outbreaks [9], and ambiguity about perceived seriousness at the source or destination for tourism or leisure [10] while avoiding international travel for leisure. Till the year 2019, China was accounted as the biggest outward tourist segment in the international market.

This study further stretches the stance of Saneinia, et al. [11], who argued that the VR-tourism experience could be the future of the tourism industry if promoted in a well-timed manner. Specifically, the current study is addresses (RQ1) how media exhaustion and information overload in the post-truth era contribute to shaping the Chinese population’s international traveling avoidance behavior, (RQ2) how the contextual factor of having a health crisis at the source and the destination simultaneously contributes to predicting the future of outward tourism for leisure in the Chinese population, and (RQ3) how the degree of innovation readiness in terms of information and technology can help to map future trends in the Chinese outward tourism industry in the post-COVID-19 era. Thus, the current study can be counted as significant and valuable, as it highlights factors which can predict resistance to outward tourism in the post-COVID-19 era. The study is intended to underline the role of the resources’ self-efficacy and literacy, which can be predicated as the crucial determinants of tourists’ readiness to adapt to changes [12]. The rest of the paper is arranged as follows: first, we conduct a comprehensive literature review, which is followed by the theoretical modeling and hypotheses declaration. The next section describes the methodology used in this study, which further leads to the findings and analysis of the collected data. In the latter half of the manuscript, the implications of the current study are discussed in detail, along with the possible future directions that the current study proposes.

## 2. Literature Review

The tourism industry played a vital role in boosting the world’s economic connectedness before the emergence of COVID-19, as it used to contribute more than 10% of the world’s GDP and offer 320 million jobs worldwide. With its multifaceted consequences, it also assisted in stimulating economic activities in the economic sector [13]. The demand for tourism has been affected by many unpredictable facets such as natural or man-made disasters, i.e., earthquakes and pandemics. In the historical view, several economic and financial catastrophes dangerously impacted tourism in many parts of the world [14]. For instance, in the years 2007–2008, The great economic crisis had a profound effect and led to significant economic upheaval in several nations, including well-known southern European vacation destinations (namely including Spain, Italy, Portugal, and Greece [15]). Moreover, political instability is also noted as a critical factor in the affected tourism industry; i.e., the annexation of Crimea and the civil war in eastern Ukraine have unequivocally depreciated the growth of the tourism industry in Ukraine and Crimea [16]. Tourism in North and Western Africa was massively hit by the emergence of Ebola during the years 2013–2016. During the Ebola outbreak, the African Cup of Nations (AFCON), the continental football tournament which was scheduled to be held in the first quarter of the year 2015 in Morocco, decided to pull out because of the Ebola outbreak [17]. Existing literature explores the effects of dengue, Ebola, yellow fever, and malaria on travelers arriving in affected countries. According to reports, such illnesses significantly reduce tourist arrivals; particularly, malaria risk in a country leads to 47% fewer tourists arriving. The travel, leisure, and tourism sectors were directly affected by SARS in China [18]. The aviation sector was also severely impacted by SARS. TravelSky, the Chinese airline computer reservation system (CRS), processed fewer reservations for Chinese commercial flights and foreign and regional airlines for the month of April alone, falling by around 10% and almost 20%, respectively [16]. The avian flu outbreak had an impact on the tourism sector as well; the Asia Pacific area had a reduction of at least twelve million foreign tourists [19].

The above-given literature concludes that pre-COVID-19 crises had a devastating impact on the economic, political, and tourism industries. However, the impact of COVID-19 was unprecedented, but also influenced the socio-economic status of individuals. Socio-economic activities were massively affected in both developed and developing countries as a result of confining measures on travel [15]. Shrestha, et al. [20] stated that, in the year 2019, 4.5 billion tourists travelled by airlines, and this number decreased to 2.2 billion during the pandemic. Fotiadis, et al. [21] illustrated that the pandemic also massively impacted supporting industries of tourism, such as the food industry. Additionally, Ivanov, et al. [22] observed the direct impact of the community exposed to the pandemic in terms of its responses to policy, combating mentioned psychological distress and mental health. The existing literature on COVID-19’s impact on the tourism industry reveals that several ex-ante research initiatives have argued before and during the pandemic, and limited studies have been conducted on the ex-post pandemic. Thus, based on the above discussion, it can be stated that there is a dire need to explore the ex-post pandemic impact on tourism.

In the post-COVID-19 era, responsible stakeholders are increasingly considering the use of ICT-based media-rich environments, i.e., providing virtual travel tools and virtual tourism activities to satisfy tourism needs [23]. When the entire world halted its commercial operations, digital tourism provided individuals with a chance to escape isolation [24]. Due to the pandemic crisis, more individuals are willing to try alternate technologies, i.e., Metaverse [25]. Go and Kang [26] stated that the deployment of a Metaverse in the tourism and hospitality industries is becoming increasingly popular among the general public. Given that, digital tourism is an essential addition to the industry, as it can be considered the future of the tourism industry [27].

The COVID-19 pandemic has set new norms and paradigms, especially in the post-truth era. Post-truth is a new phenomenon that has put more emphasis on the conditions of truth than on absolute truth. A world of disputes and paradoxes has resulted from the post-truth concerning COVID-19 [28]. It has had an impact on the political systems of numerous nations, causing the suspension of legislative and parliamentary activity, the exile and demise of numerous leaders, and the postponement of elections. Additionally, this has impacted religious activities such as pilgrimage. In the COVID-19 walk, critics have looked at several theoretical perspectives and barriers to sustainable tourism [29]. In this pessimistic environment, digital tourism can be seen as an opportunity. The tourism sector is becoming more flexible as a result of technology [30]. People have received a lot of assistance from technological professionals in the post-COVID-19 period. Numerous studies have examined the public’s confidence in technology, eagerness to connect, and openness to modifying their opinions toward it. People are increasingly aggressively leveraging IT resources for virtual tourism, bypassing privacy concerns in order to get a more significant technological benefit [30]. Unforeseen and sudden disasters are unpredictable, and the fear of these crises, as well as communication to repair the trust among the general population, is another challenging task for the tourism industry.

### 2.1. Theoretical Modeling

In terms of the behavior modeling to understand public readiness for tourism, several theoretical models can be noted as references. For instance: Cahigas, et al. [31] extended the Theory of Planned Behavior (TPB), underlining the conceptualization to determine the factors that influenced Indonesian travel to Bali during COVID-19. Stylos, et al. [29] extended the Affective Events Theory (AET) to examine tourist attitudes and behavior with the goal of better understanding the travelers’ on-board bus tour experience to strengthen the role of affect in tourism management. In light of the COVID-19 pandemic, [32] The Health Belief Model (HBM) and Value-Belief-Norm (VBN) were integrated to provide a theoretical foundation for a decision-making strategy for preventive travel. Ohnmacht, et al. [33] developed an integrated model based on the TPB and HBM and measured general risk-taking behavior in the case of COVID-19. Nazneen, et al. [34] examined the impact of COVID-19 on travel avoidance concerning the protection motivation theory (PMT). To understand health-centric behavior, the Health Belief Model (HBM) can be considered one of the most interesting theoretical stances, as it offers a holistic view of persuasive psychological understanding [35]. It explains and predicts individual understanding of health-related concerns. The HBM was initially developed by Becker (1974) and modified by Rosenstock in the 1990s. It has been studied in the existing literature to map public health-centric behavior, i.e., smoking [36], dental [37], and influenza [38]. In recent years, the HBM has been adopted in several research initiatives to map concerns and public health protective behavior during the time of the pandemic. By adopting the HBM, [39] categorized social media content uploaded during the COVID-19 outbreak, concentrating on information about the physical distancing procedures recommended by public health authorities. Similarly, Keren, Siddiquei, Anwar, Asmi and Ye [35] used media self-efficacy and scientific efficacy along with the HBM to map the health-protective behavior in times of pandemic crisis. As the purpose of the current research was to emphasize health crisis-based situations, the HBM was therefore adapted. Specifically, along with the HBM’s fundamental factors, i.e., seriousness and perceived threat (fear) of COVID-19, the role of information and its related factors were considered while defining benefits, barriers, and cues to act. Moreover, self-efficacy in terms of information literacy was proposed to extend the novelty of the adapted theoretical stance.

### 2.2. Hypothesis Development

#### 2.2.1. Perceived Seriousness: At Source and Destination

In the context of the HBM, perceived seriousness is defined as higher possibilities of being exposed to any health danger prompt people to take any health preventive activity, Researchers [40,41] mentioned that People are compelled to think about the possible negative outcomes if the preventive measures are disregarded by the seriousness and perceived health risk of the issue. In the tourism industry, [41] stated that Chinese tourists usually avoid travel plans in case of health concerns and the seriousness of health crises. Samdin, et al. [42] mentioned that females and elderly ones, particularly, prefer to cancel their travel plans when they feel the tourism destination is unsafe to visit. Likewise, if the seriousness of the pandemic is high at the source (home country), travelers prefer to avoid international traveling. Moreover, Lebrun, et al. [43] demonstrated that The COVID-19 epidemic has changed how travelers feel about their intended trip distances (proximity tourism). Furthermore, during the COVID-19 pandemic, onsite traveling became difficult, opening opportunities for virtual tourism [2]. The current research is unique, as it examined the role of the seriousness of health crises at the source and destination simultaneously (as a contextual factor). Hence, the current research proposed the following hypotheses:

**H1:**  *Pandemic seriousness at the source significantly influences individuals’ intentions to avoid international travel for leisure.*

**H2:**  *Pandemic seriousness at destination significantly influences an individual’s intentions to avoid international travel for leisure*.

#### 2.2.2. Fear of New Pandemics Outbreak

The literature indicates that fear of being exposed to a health crisis as a threat is identified as an individual’s beliefs about vulnerability to disease and the degree of seriousness of the disease [44]. In tourism-related research, perceived threat is the degree to which a prospective traveler feels secure and at ease when away from home [45]. Luo and Lam [46] have proposed that a higher degree of fear decreases the possible intention to travel. Travelers may change their destination, adjust their conduct while traveling, or get more information before going if there is a possible risk associated with that location [44]. Moreover, in the post-truth era, the information became prejudiced, and social media became a potential threat [28]. The emergence of new variants, i.e., Omicron and Monkeypox, increased the level of uncertainty among individuals [47]. According to the researchers, as the degree of uncertainty increases, the outcome is that the physical traveling intentions of tourists decrease. Physical traveling intentions can also possibly be switched towards virtual tourism, as underlined by [1]. Hence, the current research proposed the following hypothesis:

**H3:**  *Perceived fear of new pandemic outbreaks significantly influences the individual’s intentions to avoid international travel for leisure*.

#### 2.2.3. Perceived PPE Ineffectiveness

Perceived benefits refer to one’s perceptions of the effectiveness of the recommended action to lessen the risk or the severity of an impact [35]. Beliefs about the benefits of protection can have a favorable impact on behaviors that promote health [48]. Keren, Siddiquei, Anwar, Asmi and Ye [35] stated in their study that the benefits of being protected were one of the most significant constructs during COVID-19. The use of adequate PPE, i.e., wearing masks and hand sanitizing, is only one of a few infection prevention and control (IPC) strategies that are essential for reducing the risk of COVID-19 infection. Joudeh, et al. [49] identified the positive attitudes toward the use of PPE, considering it important and useful in limiting the spread of the virus. An individual will be more inclined to engage in a certain conduct if they believe it would benefit them more. People may consider undertaking PPE as the benefit of being protected, so a severe outcome of the health crisis can be avoided [50]. In the current study, the authors deliberately measured the perceived ineffectiveness of the health-protective measures, which helps to underline the perceived benefits of being protected in inverse order. Hence, the current research proposed the following hypothesis:

**H4:**  *Perceived PPE’s ineffectiveness significantly influences the individual’s intentions to avoid international travel for leisure*.

#### 2.2.4. Information Overload in the Post-Truth Era

The definition of perceived barriers is one’s perception of the financial and emotional consequences of the suggested activities. [51]. A person’s perceptions of the boundaries of engaging in a suggested activity, or a treatment that may be “inconvenient, costly, and difficult to do”, are known as perceived barriers [33]. In the context of the HBM, perceived barriers are behaviors that prevent individuals from performing health-centric actions [52]. In the post-truth era, a flood of information exists in our surroundings, and it is difficult to differentiate between false and true information [53]. The literature argues that information overload becomes a hurdle in decision-making in the case of COVID-19 [54]. With COVID-19 and tourism as the context, perceived barriers (including in the form of information overload) and information in the post-truth era significantly influence the decisions of individuals to travel [55], as information is loaded with conspiracies, misinformation, and myths [52,56]. Hence, the current research proposed the following hypothesis:

**H5:**  *Information overload in the post-truth era significantly influences the individual’s intentions to avoid international travel for leisure*.

#### 2.2.5. COVID-19 Media Reporting Exhaustion

Cues to action (in the HBM) refer to the events or experiences which provide circumstances for an individual to take health protective measures. Social media and the internet are seen as resources for finding health related information. [57]. During the COVID-19 pandemic, the risk of misinformation related to the pandemic forced the World Health Organization to declare that “the world is fighting Pandemic along with infodemic” [58]. Social media sites both facilitate and spread false and inaccurate information about COVID-19 [59,60]. In the tourism context, [61] identified that to stay informed about disease development, travelers rely on news media and cues. As the world also experiences information overload during the ongoing pandemic crisis, individuals find it difficult to identify trustworthy sources from false ones; as a result, because of reliance on social media, people started to trust fake news and implement that in their lives [62]. Hence, the current research proposed the following hypothesis:

**H6:**  *Media reporting stress (as a part of cues to act) significantly influences the individual’s intentions to avoid international travel for leisure.*

#### 2.2.6. Information Literacy (as a Soft Aspect of Self-Efficacy)

The HBM was adapted by several researchers with an extended view to maximize its explanatory power. In the current research initiative, the authors extended the use of self-efficacy in the context of the information era, where information literacy is taken into account. Self-efficacy is the conviction that one can carry out the desired conduct in spite of perceived barriers. In this context, it has been explained that self-efficacy affects adherence to safety precautions that reduce needless travel [61]. According to research, false information spread online has a greater impact on individuals than news that is supported by facts, which causes individuals’ information literacy (efficacy) to seek information [12]. Information literacy is described as a collection of abilities to find, comprehend, assess, and use information [12]. Recently, Nawaz, et al. [63] examined the moderating role of “Information Literacy”, as it can enhance consumers’ behavior. In light of the above argument, the current study focuses on information literacy providing self-efficacy to decide in the case of traveling intentions. Therefore, this study proposed the following hypothesis:

**H7:**  *Information literacy significantly influences the relationship between (a) perceived PPE ineffectiveness, (b) information overload in the post-truth era, and (c) media reporting stress with the individual’s intentions to avoid international travel for leisure*.

#### 2.2.7. Attitude toward Digital Tourism (as the Hard Aspect of Self-Efficacy)

Several studies have investigated a vast variety of individual factors that are associated with intentions to avoid traveling internationally for leisure, i.e., the fear of catching the virus, seriousness of the virus, restrictions on travel, and vaccine requirements [49]. Among these factors, an individual’s readiness to adopt alternate choices also includes the possibility of switching to digital tourism [11]. The literature also argues that digital tourism represents an appealing alternative to conventional tourism practices. In this digital era, where smartphones have altered the attitudes of individuals and made it easier to obtain information, making virtual tourism accessible to internet users, it offers a safe “seeing environment” [64]. Studies have shown that during the COVID-19 pandemic, virtual tourism obtained a tourist attraction [2]. Conner, et al. [65] assessed the moderating effect of attitude stability on behavioral relationships in their study. Based on the above-discussed arguments, this study proposed the following hypothesis:

**H8:**  *Attitude towards digital tourism significantly influences the relationship of (a) individual’s fear of pandemic outbreak, (b) pandemic seriousness at source, and (c) destination with the individual’s intentions to avoid international travel for leisure*.

**Figure 1:**
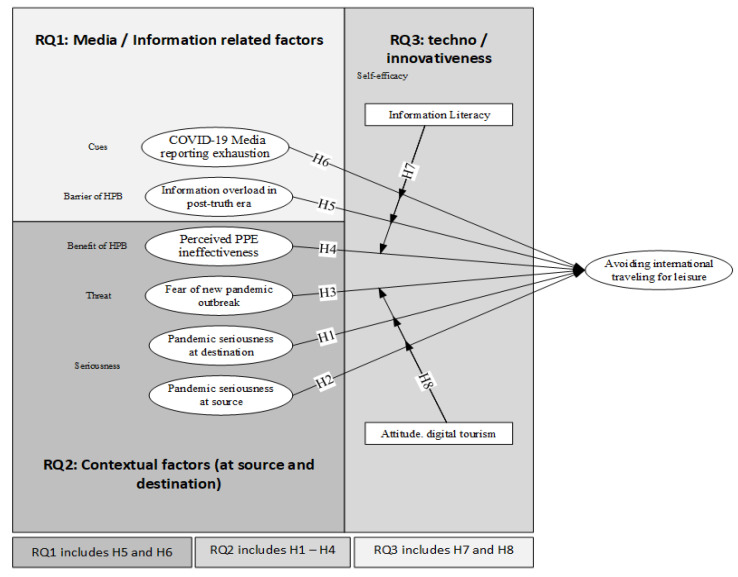
Graphical view integrating research questions and proposed hypotheses.

In the context of the current research, the operational definitions of each construct and its relevance to the existing literature are listed in Appendix A. The graphical view integrating proposed research questions and the hypotheses of the study is shown in Figure 1.

## 3. Research Methodology

### 3.1. Research Instrument

The survey concept was modified from other research to guarantee content validity. Table A2 in Appendix A lists the items used. The scale for excessive pandemic seriousness at source and destination was adapted from [66]. The scales for fear of a new pandemic outbreak and perceived PPE ineffectiveness were adapted from [67] and [68], respectively. The scale for information overload in the post-truth era was adapted from [69], while COVID-19 media reporting exhaustion was adopted from [70]. Information literacy and attitude toward digital tourism were adopted from [71] and [63]. The last scale, which is avoiding international traveling for leisure, was adapted from [69]. Based on the behavioral factors, age, gender, and education were considered as control variables. A Likert scale of 1 to 5 was used for all responses related to constructs, with 1 representing strong disagreement and 5 representing strong agreement. The instrument was originally developed in English, but the survey was collected from Chinese citizens via WeChat users, so it was necessary to translate it into Chinese; this was done by performing the back translation as described by [38] to verify the validity and reliability of the instrument. To address this issue, the authors invited three native Chinese speakers to participate in the study. These three individuals assisted with translating the English version of the survey into Chinese. Afterwards, the survey was translated back to English from its Chinese version to eliminate context-related ambiguities. To address the content validity of the study before data collection, the authors approached two experts from the Department of Communication and Technology management to review and evaluate the instrument voluntarily, as well.

### 3.2. Data Collection

A survey questionnaire was designed to collect data from the Chinese population in the third quarter of 2022. To collect data from the targeted population, a market research firm, Credamo (https://www.credamo.com (12 September 2022)), was involved. It had 1.5 million strictly censored users and could assist researchers in reaching potential participants, as observed in the existing literature. It could assist researchers in reaching potential participants through online forums or member recommendations and by rewarding study participants. Researchers in China have found it among the most valuable resources for collecting survey data [72,73]. There were several pre-check questions in the survey, including whether (1) participants had traveled internationally for leisure/tourism in the past, (2) had experienced VR experiences, and (3) hadn’t traveled abroad till now during the time of COVID-19. We focused on China, since the targeted population was rated in the top 5 nations experiencing more outward tourism. The survey comprised a cover page stating that participants were anonymous and their contributions were voluntary. In total, 1308 complete responses were collected and analyzed. The descriptive profile of the collected sample is listed below in Table 1.

## 4. Analysis and Findings

### 4.1. Measurement Model

The authors conducted an exploratory factor analysis using SPSS to ensure the validity and reliability of the collected data [74]. According to the results of the EFA, as presented in Table 2, all items’ factor loadings are above the lower cut-off value of 0.700, and there is no indication of cross-loading in adopted settings.

There was a lower cut-off value of 0.700, and there was no indication of cross-loading in adopted settings. Cronbach’s alpha, composite reliability (CR), and average variance extracted (AVE) were calculated to determine convergent validity, and the satisfactory results were concluded as reported in Table 2; each construct met the threshold values. The Heterotrait Monotrait (HTMT) and Fornell and Larcker (1981) approaches were employed to assess discriminant validity. As a rule of thumb, HTMT scores should be lower than 0.90 [75], while Fornell and Larcker’s correlation scores should be below the square root of the AVE [35]. During the external validity computation, the overall model’s discriminant reliability score was satisfactory (see Appendix A). Lastly, for saturated model fit indices were calculated, which resulted in acceptable limits as suggested by [76]. As a result of the analysis, the following fit indices were observed: Chi-square = 1085.2, degree of freedom = 324, CMIN/DF = 4.29, GFI = 0.92, AGFI = 0.90, CFI = 0.94, TLI = 0.94, and RMSEA = 0.05 (reported in Appendix A).

### 4.2. Structural Model

**Figure 2:**
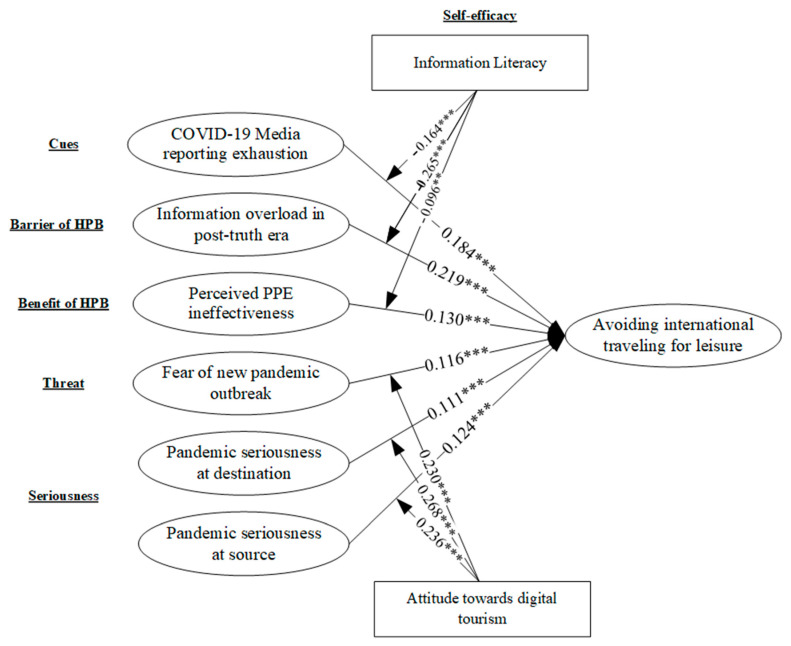
Graphical view of the structural model (proposed hypotheses). *** *p* < 0.001. ** *p* < 0.010.

Based on the satisfactory fitness indices of the proposed model, the structural model was tested. The mode fit indices are reported in Appendix A. Considering the results shown in an acceptable model fit, we proceed to calculate the path coefficient. According to the study’s results, the path coefficients calculated are significant. The graphical view of the structural model is shown in Figure 2.

The findings conclude that the seriousness of the pandemic at the source (H1: β = 0.124) and destination (H2: β = 0.11) significantly influenced the individual’s intentions to avoid international travel for leisure. Similar findings were reported by [77,78] in the case of other pandemic seriousness intensity and related behavioral studies. The fear of a new pandemic outbreak positively influenced avoidant behavior with regards to international traveling for leisure (H3: β = 0.116). The perceived PPE infectiveness was also recorded as positive with avoiding international traveling for leisure (H4: β = 0.130). It underlines that the Chinese population avoids international traveling for leisure if there is doubt about the effectiveness of the health-protective measure. Similar trends were discussed by [79]; thus, H4 was supported. Information overload in the post-truth era significantly influenced avoiding international traveling for leisure (H5: β = 0.219). It predicts that a flood of misinformation can create an uncertain situation in which differentiating accurate information is difficult. Similarly, individuals who pose information overload can be the reason to avoid international traveling for leisure, as suggested by [6,80]. The results of the study demonstrate the significant relationship between COVID-19 media reporting exhaustion and avoiding international traveling for leisure (H6: β = 0.184). They highlight that the conspiracies and misinformation related to a health crisis trigger individual traveling phobia. Karl [81] and Koh [2] also observed similar findings in politicization settings. Moreover, none of the control variables were noted as significant in the current research.

### 4.3. Moderation Analysis

The moderating role of information literacy and attitude toward digital tourism was measured with the help of the hierarchical regression model. The study examines the moderating effect of information literacy of Chinese tourists on the relationship between COVID-19 media reporting exhaustion, information overload in the post-truth era and perceived PPE ineffectiveness and avoiding international traveling for leisure. H7, positing that information literacy dampens the relationship between COVID-19 media reporting exhaustion, information overload in post-truth-era and perceived PPE ineffectiveness, and avoiding international traveling for leisure, was supported by the results (H7a: β = −0.164; H7b: β = −0.265; H7c: β = −0.096, respectively). The finding highlighted the dark side of digitalization. The proliferation of conspiracies and misinformation populates doubts in the mind of individuals, which leads to avoidance behaviors. Information overload and complexity affect cognitive dissonance. To dampen these effects, information literacy plays a vital role. Similar trends were confirmed in the recent literature [63]. In the case of attitude towards digital tourism, it was noted to positively strengthen the relationship between fear of new pandemic outbreaks, pandemic seriousness at the destination and source, and avoiding international traveling for leisure (H8a: β = 0.230; H8b: β = 0.268; H8c: β = 0.236), which is also argued by Chua et al. [82]. Diffusion of innovation in the adoption of technology positively influences individual attitudes towards alternatives to tourism, i.e., digital tourism, which corresponds with the existing literature [83]. The results in tabular format are listed in Appendix A.

## 5. Discussion

In the overall model, all hypotheses were significant and supported. The graphical representation of the model is represented in Figure 1. Our finding elucidates theory-based constructs having a relationship with individual avoidance behavior towards international traveling for leisure in the post-COVID-19 era. The finding reveals that most of the participants were in the educated segment and belonged to generation Z and millennials. Based on the RQs, as initiated in Section 1, the post-truth era is a challenging period. In our study, the findings of **RQ1** reveal that the role of media is decisive. From all the exogenous variables, the constructs that contributed most were related to media. It can be concluded that media has a strategic role in tourism and hospitality management, especially while communicating about the perceived barriers and benefits of being protected in the context of the HBM. Media has a direct and indirect impact on individuals’ behavior. During COVID-19, social media platforms played a dual role in facilitating and impeding. In this study, we found media exhaustion was the most dominant factor. The abundance of misleading information related to COVID-19 may lead to increases in anxiety, depression, and stress, which may badly influence individuals’ decision-making process. The findings of this study are in line with the existing pool of literature [9,83,84]. In the post-truth era, information and communication about effective measures are noted as crucial to shaping health protective behavior, which can be generated from several reasons: for instance, phobia, rumors of international relations, culture, national representatives, etc., strongly influence the intentions of individuals’ travel avoidance behavior. In the pre and post-pandemic era, it is difficult to determine the certainty of outbreaks, particularly in COVID-19. Several countries have experienced many waves of COVID-19, with new variants emerging in a matter of weeks—for instance, omicron, Delta, and so on so forth. Throughout, people are engaged in discussing COVID-19, the new emerging variants, and its deadly impacts on human life. In this situation, two fundamental questions arise on the effectiveness of taking all the measures during the COVID-19 outbreak and the ability of new outbreaks to cope with the existing vaccine. All these concerns appeal to different fears that trigger depression, anxiety, and mental illness. The findings of this study are in line with [85], which revealed that the seriousness and fear of new pandemics led Chinese people to avoid traveling internationally and prefer domestic travel for leisure. We conclude on the basis of our findings that it is the second most dominating factor that shapes individual international travel avoidance behaviors. While exploring the findings of **RQ2,** the current study reflects that in the tourism and hospitality industry, taking contextual factors into consideration is an important aspect. Several studies discussed pandemic seriousness at destination in the tourism industry, but pandemic seriousness at source is still a loophole to explore. Though the lack of facilities at the source has been studied in the existing literature [77,86], none of the studies have considered and compared pandemic seriousness at the source with destination and its outcomes. To address this gap, the current study took the initiative to consider pandemic seriousness at its source and highlighted its vital effects on travel avoidance internationally. Interestingly, our findings determine that source is a more serious concern as compared to destination because it is a first-hand experience of an individual, as shown in the results of this study. **RQ3:** In the era of digitalization, people’s readiness towards awareness and acceptance of technology is a matter of concern for predicting future perspectives. Therefore, the current study took these factors as moderating constructs. The study inquired about personal innovation and information adaptability as self-efficacies (technology-related cognition was considered as he soft side, whereas attitude towards digital tourism was considered as the hard side of technology). In the case of information literacy, the degree of innovation in the post-truth era helped in coping with media challenges. On a positive note, in the tourism and hospitality industry, technology innovation readiness and attitude toward digital tourism were noted while coping with pandemic seriousness. The study revealed a positive individual attitude toward digital tourism. The findings of this study are in line with the existing literature [2]. COVID-19 bans on international travel for leisure lead the Chinese population to alternate options and sources of leisure and tourism, i.e., augmented-reality-based environment.

## 6. Conclusions

This current study initiative proposed to extend the health behavior model to conceptualize the international travel avoidance behavior of Chinese people for leisure. This study is the first to underline the role of media, pandemic, and contextual attributes. Moreover, the roles of media exhaustion and information literacy were also researched further using the existing HBM in the current study. The findings of this study reveal that the role of media was the greatest contributing factor, since media has a direct impact on individuals’ behavior. However, the findings of this study conclude that information literacy aids with mitigating media exhaustion and helping individuals in decision-making. Uncertainty of new variants created fear of being infected among individuals; thus, the valuable findings of this study underline that the seriousness and fear of new pandemics led Chinese people to avoid traveling internationally. The findings also explore source being more serious as compared to destination. Additionally, this study reveals that COVID-19 bans on international travel for leisure influenced individuals’ positive attitudes towards digital tourism. Individuals were found to be more engaged with VR and Metaverse to satisfy their travel needs.

### 6.1. Theoretical Practical

The current study signifies the roles of health priorities and health-related information literacy while conceptualizing the health-centric behavior of Chinese international travelling for leisure. The current study puts forward several theoretical implications for researchers and policymakers. The theoretical and practical implications of this study are as follows.

The theoretical implications of this study are as follows. *First*, the most important theoretical contribution of the current research is that this research studied the HBM, one of the widely applied theories in health-centric behavioral study, as it can predict the health-centric behavioral intentions of individuals. This study examined the HBM through a different lens, mainly divided into three attributes, i.e., the role of social media, the role of pandemics, and contextual (source and destination) attributes. In the existing literature, the HBM has been widely used and extended; therefore, this study also revised and extended the view of the HBM as adapted by [35], which discussed media self-efficacy as a moderator. However, this current study researched the standpoint of media self-efficacy as an exogenous variable. As Jones, et al. [78] argued, the HBM has flexibility and liberty to explore factors other than fundamentals that help in shaping the behaviors of an individual. Continuing his view, the current study explores contextual attributes as a novel contribution. As the findings of this study reveal, the role of media was the greatest contributing construct. Thus, this study concluded that media plays a significant role in tourism and hospitality management. *Second*, in the pre- and post-COVID-19 times, it was difficult to determine the uncertainty of outbreaks and the uncertainty of new variants of COVID-19, and increasing fear of related outbreaks has been explored descriptively in the existing literature. However, none of the studies explored fear of COVID-19 variants in quantified modeling. The current research examined people’s fears of new variations as a perceived threat in the hood of HMB as a theoretical stance. *Third*, this current study shaped the role of information and technology in the post-COVID-19 pandemic in terms of how it impacts contextual and media-related factors. In the case of information literacy, it gives firm support to avoiding cognitive dissonance. Our findings demonstrate that information literacy has a significant influence on individuals in changing their behavior and dampening the dark impact of media exhaustion, information overload, and perceived PPE ineffectiveness in the post-truth era. These outcomes primarily raised more attention to coping with conspiracies and fake news. Interestingly, information literacy is noted as an antidote in mitigating the risks and challenges of the post-truth era while dealing with such a health crisis. During the pre-COVID-19 era, people became more open to adopting technology and gained more interest towards digital tourism. This study also concluded that attitude toward digital tourism and readiness to accept technology innovativeness can lead to an opportunity to drive new branches in the tourism industry, where technology innovation can play its strategic role.

### 6.2. Practical Implications

The practical implications of the study are drawn as follows. First, like other international affairs, COVID-19 also affected international relationships among countries. Politicization in terms of public opinion badly influenced pre-COVID-19 national and international relations worldwide during the pandemic, i.e., in the case of China. The current study underlines that pandemic seriousness at source and destination holds a different degree of influence over Chinese tourists’ outward traveling intentions. Thus, to regain public trust in the Chinese masses, internationally prominent tourist spots need contextual tourism marketing companies in the future. Post-COVID-19 concerns should be tailored according to cultural and contextual manners. For future tourism promotion, countries must keep in mind not only the source, but also the link, and synchronize source and destination. While designing marketing campaigns, tourism industries should focus on customization. Tourism and hospitability authorities should try to focus on tailored strategies to increase demand and ensure a comfortable environment to tourists. COVID-19 has affected everyone around the globe; to regain a stable political ecosystem among countries, all the actors must support each other to flourish together. Otherwise, it will be challenging for countries to survive, especially in the tourism industry. *Second*, this study draws conclusions regarding the significant role of information overload and media exhaustion while reporting. During COVID-19, it was seen that an abundance of fake and true information proliferated on social media, which created exhaustion among individuals. When organizations implemented policies related to COVID-19, they ignored the role of media exhaustion and information overload, which initiated conspiracies and misinformation. To cope with this dark side of digitalization, myths, misperceptions, and misunderstandings among the masses must be eliminated through tourism-related communication internationally. *Third*, we are stepping into the *Digi-world* (digitalization); interestingly, the seriousness of the contagious nature of the COVID-19 pandemic opened up the window of opportunity for VR-based tourism to flourish. Thus, the study encourages interested stakeholders to get involved in a contemporary fashion where augmented reality, virtual reality, Metaverse, and mixed-reality-based appealing environments can be captioned as part of the future of tourism industries worldwide. To promote VR tourism, authorities should pay attention to and design a face-lifting environment, resources hardware, soft resources, and policies to identify and integrate stockholders.

### 6.3. Future Studies

Even though this study provides interesting insights, its limitations should be acknowledged, as they may open up several opportunities for future researchers. First, in the post-truth era, media plays a negative role in influencing individuals’ decision-making because of information overload, which creates media exhaustion. To elaborate on the role of media explicitly, future research should be investigating which kind of information over social media is crucial in the post-COVID era. Second, this study found that information literacy plays a significant role in mitigating the dark side of media exhaustion; therefore, authorities should promote information literacy by communicating scientific knowledge on social media to inform individuals with accurate information. Third, the current research investigated the attitude of individuals toward digital tourism. To date, none of the studies performed in the context of the HBM have measured switching intentions towards VR-digital tourism. In other words, a future study can be performed on the attitudes of individuals towards switching from physical tourism to virtual-based tourism in the context of the HBM.

## Figures and Tables

**Table 1:** Profile of the collected sample.

Characteristic	Detail	Freq.	In Percentage
Gender	Male Female	683 625	52.22 47.78
Age	Under 25 25–40 Above 40	594 417 297	45.41 31.88 22.71
Education (last attended)	Secondary School Higher Secondary School Vocational College/College University	169 178 487 474	12.92 13.61 37.23 36.24
Have you ever tried any kind of VR experience for entertainment	Yes No	1034 274	79.05 20.95

**Table 2:** Loadings and reliability testing of the constructs.

Construct	Items	Factor Loadings	Cronbach Alpha	Composite Reliability	Average Variance Extracted
COVID-19 Media Reporting Exhaustion (CME)	CME1 CME2 CME3 CME4	0.886 0.815 0.794 0.813	0.846	0.897	0.685
Information Overload in Current Post-Truth era (IO)	IO1 IO2 IO3 IO4	0.850 0.798 0.773 0.784	0.816	0.878	0.643
Pandemic Seriousness at Destination (PSD)	PSD1 PSD2 PSD3	0.872 0.858 0.828	0.836	0.889	0.752
Fear of New Pandemic Breakout (FNPO)	FNPO1 FNPO2 FNPO3	0.879 0.830 0.855	0.816	0.891	0.731
Perceived PPE Ineffectiveness (PPI)	PPI1 PPI2 PPI3	0.894 0.844 0.863	0.815	0.901	0.752
Pandemic Seriousness at Source (PSS)	PSS1 PSS2 PSS3	0.848 0.822 0.841	0.788	0.875	0.701
Avoid International Travel for Leisure (AITL)	AITL1 AITL2 AITL3	0.874 0.840 0.811	0.795	0.880	0.709
Information Literacy (IL)	IL1 IL2 IL3 IL4	0.902 0.796 0.800 0.790	0.840	0.894	0.678
Attitude towards Digital Tourism (ADT)	ADT1 ADT2 ADT3 ADT4 ADT5 ADT6	0.889 0.783 0.786 0.800 0.769 0.776	0.888	0.915	0.645

## Data Availability

Data will be available on reasonable request.

## Appendix A

**Table A1:** Operational definition and relevance with the existing literature.

Variable Name	Operational Definition	Findings of the Recent Literature
COVID-19 Media reporting Exhaustion (CME)	Media reporting exhaustion is a psychological reaction to the sensation of fatigue brought by media in such worrying situations as COVID-19 [70].	Luo and Lam [46] examined the relationship between media exposure to COVID-19 and acute stress response. Results of this study reveal that longer media. (print/social) exposure time was significantly related to high acute stress. Findings confirm the relationship between indirect media exposure to pandemic events and acute stress. Yang, Chen, Heidari and Gandomi [77] proposed a model portraying the interrelation between China’s country image, destination image, and travel intention in relation with misleading media reporting. Zhang, Huang, Chen and Zhao [83] proposed a conceptual model to test the relationship between media reporting, outbound travel intentions, risk perception, and preparations. The findings reveal that media coverage significantly influences the affective risk perception and outbound traveling intention.
Information Overload in Current Post-Truth era (IO)	A condition or situation in the post-truth era in which an individual is unable to effectively evaluate and make use of all the available information. It occurs when processing accedes to one’s capabilities [84]	Jia, et al. [87] explored the relationship between information overload and message fatigue toward COVID-19 prevention decision-making of Americans and Chinese people. The findings conclude that information overload and message fatigue were lower in Chinese people while making decisions related to COVID-19 prevention. The researchers of Zhang, et al. [80] investigated whether environmental concerns for tourism and hospitality were important in the light of information and choice. The findings show that the major obstacle to changing tourism behavior is making it a higher priority, which is a challenging task given the environmental issue overload that most people have to cope with. Tan and Kuo [5] highlighted the most ignored effect of information overload uncertainty in the context of tourism. The results of this study conclude that overload uncertainty increased undesirable outcomes such as regret and weakened the desirable outcome.
Pandemic Seriousness at Destination (PSD)	Pandemic seriousness at the destination is the perceived severity of COVID-19 or related outbreaks at the destination where one desires to visit; as a result, individuals avoid traveling [88].	Han, Che and Lee [85] examined the relationship of Korean tourist traveling avoidance behavior toward China as a traveling destination during COVID-19. This study reveals that risk perceptions and negative predicted emotions were significant factors in avoidance behavior. The findings also show that destination attachment lowered the influence of risk perception related to avoidance behavior. Morar, et al. [89] investigated travel behavior and psychological factors during the pandemic changing tourist preferences due to traveling restrictions at the destination. Results show individuals preferred less travelling in groups, especially foreign travelling. Individuals were more cautious when they perceived high risk of infection at the destinations; thus, higher fear at destinations lowered intentions to travel.
Fear of New Pandemic Breakout (FNPO)	Fear stimulates anxiety and individuals’ response toward perceived risk. The uncertain evolution of outbreaks with high rates of contagion and mortality fosters individuals’ fear, implying negative consequences for subjective well-being [67].	Qureshi, et al. [82] conceptualized identifying knowledge perception and fear of new emerging variants of Omicron among the general public. Their study found that people believe that new variants are more fatal as compared to COVID-19, and the level of fear was higher among respondents. Zheng, Luo and Ritchie [1] explored the impacts of trust, fear, and perceived threat in post-pandemic travel decisions. This study suggested that fear can lead to change in human behavior and travel avoidance during public health crises. People deal with dangerous situations by avoiding travel as a defensive emotional response in health crises. Zheng, et al. [90] explored the factors that cause pandemic travel fear in the public and the way people impose self-protection or coping related to travel. The findings reveal that threat severity and susceptibility caused travel fear, which led to protection motivation and travel behavior.
Perceived PPE Ineffectiveness (PPI)	Individuals’ attitudes regarding not using PPE because they believe it can be ineffective, difficult to use, and subject to an offense-safety point system [68].	Mallinas, et al. [91] examined psychological and demographical factors that influence attitudes towards mask-wearing during COVID-19. Two studies differentiated pro-mask from anti-mask attitudes. The findings show that psychological reactance was associated with anti-mask attitudes, above and beyond political ideology and age. Reactance was associated with anti-mask use attitudes, whereas fear, trust of healthcare professionals, empathy, and perceived normativity were associated with pro-mask use attitudes. Taylor and Asmundson [79] proposed a comprehensive investigation of anti-mask attitudes during the COVID-19 pandemic. According to the findings, people who don’t wear masks and have negative attitudes towards wearing masks believe that masks are ineffective and that they are being forced to wear masks.
Pandemic Seriousness at Source (PSS)	Pandemic seriousness at source is the severity of COVID-19 in the home country, which prevents individuals from travelling internationally and causes them to prefer to stay at home [66].	Dzhambov, et al. [92] revealed in their study that, during the seriousness at the source, stay-at-home strategies prohibited outdoor activities for an unprecedented period of time, and this caused fear, uncertainty, increased depression, anxiety and insomnia, etc. Greenery, both indoors and outdoors, has been considered a healer to mitigate depression; in line with this, the current study found that students who spent most of their time at home during COVID-19 experienced better mental health when exposed to more greenery.
Avoid International Travel for Leisure (AITL)	The avoidance behavioral change in one’s leisure travel plans shaped by the health -related risks and fear of contracting the novel COVID-19 disease [93].	Chua, Al-Ansi, Lee and Han [93] addressed the systematic relationship between negative affect, health risk perception, mental well-being, perceived uncertainty from a U.S. tourist perspective, as well as their role in affecting travel behavior and avoidance behavior towards global destinations seriously hit by the pandemic. The findings demonstrate that negative affect as a result of COVID-19 significantly influenced perceived health risk, which in turn induced mental well-being and perceived uncertainty. However, mental wellbeing predicted attitude towards international travel and avoidance behavior. The researchers of Barazi, et al. [94] investigated the impacts of COVID-19 on tourists’ destination choice using six factors. The results showed that Saudi tourists not only avoided international travel but domestic travel as well because of the severity of the pandemic, given that risk of dying was higher in females. Nazneen, Xu, Din and Karim [34] conceptualized protection motivation theory to investigate the direct and indirect relationship between COVID-19 impacts and travel avoidance. This study indicates the positive relationship between COVID-19 impacts and travel avoidance. During the pandemic, individuals avoided international travel for leisure because of risk perception, health, and safety.
Information Literacy (IL)	Information literacy is proficiency level in finding, acknowledging, interpreting, and adequately utilizing online information in an ethical way [12].	Kruijt, Meppelink and Vandeberg [12] explored strategies such as critical thinking to help individuals discern true news from fake news on social media during COVID-19. The findings suggest that information literacy played a moderating role in combating the issue. Nawaz, Anwar, Zhou, Nawaz, Dong and Asmi [63] proposed the crucial role of health concerns in consumer willingness towards GMF food products. The results conclude that information literacy plays a moderating role in enhancing consumers’ willingness to consume GMF by decreasing health concerns. Borah, et al. [95] examined the association between media literacy and attitude of individuals towards recommended COVID-19 health-centric behavior. This study also examined the moderating role of media. The outcomes represents that media was adversely associated with perceived behaviors, and literacy of media was positively associated to individuals’ willingness to perform COVID-19 related health behavior. Media literacy can reduce the negative impact of media on individuals.
Attitude towards Digital Tourism (ADT)	The attitudes and actions of people toward using new technology, as well as their readiness to accept digital tourism: “a video image sequence that represents a simulation of an actual site” [23].	Akhtar, et al. [88] explored the attitude and future development of digital tourism after the pandemic crisis. The findings reveal that mass tourism aided through virtual tourism in the post-COVID-19 era is a valuable option. This study also reveals a positive individual attitude toward digital tourism. Talwar, et al. [96] proposed a sequential mechanism using stimulus organism response theory (SOR) to theorize VR tourism as a sustainable solution for the future. The outcomes confirmed the positive attitude towards digital tourism and continuance intentions in post-COVID-19 time. Zheng, et al. [97] explored the impact of digital tourism on Mental Imagery Processing (MIP) and cognition and emotion to identify the impacts of MIP on future behavioral intentions to visit actual tourist attractions. The findings show the influence of MIP on emotion and cognition, which affects future visitation. MIP decreased interest because of negative emotion, but inspired attitudes towards visiting through learning.

**Table A2:** Constructs, instruments, and adapted sources.

Variable Name	Instrument	Source
COVID-19 Media Reporting Exhaustion (CME)	I feel drained while following COVID-19 related news in print or electronic media. I feel tired while following COVID-19 related news in print or electronic media. Using print or electronic media to get updates on the ongoing pandemic crisis is a strain for me. I feel burned out while following COVID-19 related news in print or electronic media.	[72]
Information Overload in Current Post-Truth era (IO)	The excessive amount of information about the pandemic in this post-truth era, travel restrictions, and their related impacts makes me feel… … distracted … overwhelmed … that it is a burden to handle … that only a small part of the information is relevant to my needs	[98]
Pandemic Seriousness at Destination (PSD)	It is very likely that I will be infected with the new coronavirus/new wave if I travel to the desired destination for leisure. It is very likely that I will be infected with the new coronavirus/new wave if I travel to the desired destination for leisure, as compared to others in my same age group. It is very likely that I will be infected with the new coronavirus/new wave if I travel to the desired destination for leisure, as compared to others of my same gender.	[66]
Fear of New Pandemic Breakout (FNPO)	It is a great risk to have a new variant of COVID-19 or any similar kind of pandemic crisis. It is likely that we will have a new variant of COVID-19 or any similar kind of pandemic crisis. The chances of having a new variant of COVID-19 or any similar kind of pandemic crisis are high.	[67]
Perceived PPE Ineffectiveness (PPI)	In the time of the recently ongoing pandemic crisis, using PPE is… … is not a worthy idea, as it can not protect me 100%. … is a not-so-wise idea, as it can not protect me 100%. … what I personally dislike, as it can not protect me 100%. … not convenient, as it can not protect me 100%. (DROPPED)	[68]
Pandemic Seriousness at Source (PSS)	It is very likely that I will be infected with the new coronavirus/new wave while staying in my country. It is very likely that I will be infected with the new coronavirus/new wave while staying in my country as compared to others in my same age group. It is very likely that I will be infected with the new coronavirus/new wave while staying in my country as compared to others of my same gender.	[66]
Avoid International Travel for Leisure (AITL)	In the near future… … I will not think about planning any international trips for leisure. … I will think about alternate ways for leisure instead of internal traveling. … If I could, I would travel internationally for leisure for an indefinite period of time.	[71]
Information Literacy (IL)	I know how to organize information and its details in routine life. I am aware of sources to look for required information in routine life. I know where to find information when needed in routine life. I can understand the information type and importance of the information source I need in routine life.	[71]
Attitude towards Digital Tourism (ADT)	In my perception, using digital tourism can be… … exciting … diverse … attractive … unique … appealing … innovative	[99]

Non-response bias testing.

Since we collected cross-sectional data through a single instrument, common method bias (CMB) may be present in the response. To avoid CMB, the current study employed three quantitative approaches for behavioral modeling. First, Herman’s single factor biases approach was implemented to measure the maximum variance among the proposed constructs, as suggested in the existing literature Hair Jr, et al. [100]. Noticeably, in the overall model, the maximum variance by single factor observed was 25.34%. Second, common latent factor (CLF) testing was also performed on the adapted instrument, as followed by Keren, Siddiquei, Anwar, Asmi and Ye [35]. The standard regression score of models with or without CLF was observed, with no significant difference greater than 0.20. Thus, the quantified result of the study claims no CMB. Third, to determine multi-collinearity, the variance inflation factors (VIF) score was recorded, noting that less than three is acceptable [101]. The tabular format of the VIF scores is listed in Appendix A. Thus, there were no multi-collinearity concerns in the current study. Additionally, to ensure the reliability of the collected data, non-response bias was also measured when the initial and latter parts of the collected responses were compared by using the chi-square test. The findings reported no significant differences between both data groups. Thus, the study can be claimed to be free of non-response bias-related issues.

**Table A3:** Variance Inflation Factors analysis.

Indicator	CME	IO	PSD	FNPO	PPI	PSS	AITL	IL	ADT
CME1	2.730								
CME2	1.929								
CME3	1.739								
CME4	1.758								
FNPO1				2.188					
FNPO2				1.741					
FNPO3				1.750					
PSD1					2.247				
PSD2					1.643				
PSD3					1.861				
IO1		2.343							
IO2		1.569							
IO3		1.641							
IO4		1.675							
PPI1			2.495						
PPI2			1.879						
PPI3			1.884						
PSS1						1.964			
PSS2						1.526			
PSS3						1.689			
IL1								2.745	
IL2								1.755	
IL3								1.812	
IL4								1.737	
ADT1									3.080
ADT2									1.898
ADT3									1.903
ADT4									2.023
ADT5									1.821
ADT6									1.881
AITL1							2.119		
AITL2							1.664		
AITL3							1.623		

Variance inflation factors (VIF).

**Table A4:** HTMT scores.

Construct	CME	IO	PSD	FNPO	PPI	PSS	AITL	IL	ADT
CME									
IO	0.308								
PSD	0.151	0.194							
FNPO	0.300	0.343	0.139						
PPI	0.330	0.324	0.143	0.266					
PSS	0.282	0.288	0.152	0.296	0.276				
AITL	0.420	0.464	0.263	0.363	0.370	0.358			
IL	0.365	0.390	0.166	0.353	0.399	0.403	0.390		
ADT	0.390	0.492	0.142	0.377	0.387	0.376	0.472	0.319	

**Table A5:** Discriminant reliability as suggested by Fornell and Larcker (1981).

Construct	CME	IO	PSD	FNPO	PPI	PSS	AITL	IL	ADT
CME	**0.827**								
IO	0.070	**0.802**							
PSD	0.017	0.027	**0.867**						
FNPO	0.064	0.082	0.014	**0.855**					
PPI	0.078	0.074	0.015	0.048	**0.867**				
PSS	0.054	0.056	0.015	0.058	0.053	**0.837**			
AITL	0.123	0.147	0.047	0.088	0.095	0.084	**0.841**		
IL	0.095	0.108	0.019	0.085	0.111	0.109	0.102	**0.823**	
ADT	0.115	0.181	0.015	0.104	0.112	0.101	0.159	0.074	**0.803**

Squared correlation and square root of AVE in the diagonal.

**Table A6:** Model fit indices recorded in the current study.

Fit Indices	Recommended Range	CFA	Model
**Chi-square**		2107.59	2126.99
**Degree of freedom**		491	493
**CMIN/df**	≤5	4.29	4.31
**GFI**	≥0.90	0.92	0.92
**AGFI**	≥0.80	0.90	0.90
**TLI**	≥0.95	0.94	0.94
**CFI**	≥0.95	0.94	0.94
**RMSEA**	≤0.08	0.05	0.05

**Table A7:** Structured path analysis of the proposed model.

	Effect	Original Coefficient	Standard Bootstrap Results				
			Mean Value	Standard Error	*t*-Value	*p*-Value (2-Sided)	Cohen’s f2
H1	PSS -> AITL	0.124	0.124	0.025	5.013	0.000	0.019
H2	PSD -> AITL	0.111	0.112	0.025	4.531	0.000	0.017
H3	FNPO -> AITL	0.116	0.115	0.027	4.371	0.000	0.016
H4	PPI -> AITL	0.131	0.132	0.025	5.228	0.000	0.020
H5	IO -> AITL	0.219	0.220	0.023	9.634	0.000	0.055
H6	CME -> AITL	0.184	0.185	0.026	7.151	0.000	0.040

**Table A8:** Description and outcome of the proposed moderating effects.

Hypotheses	Description	Coefficient and *p*-Value	Description
**H7(a)**	CME * IL → AITL	−0.164 **	IL dampens the positive relationship between CME and AITL
**H7(b)**	IO * IL → AITL	−0.265 **	IL dampens the positive relationship between IO and AITL
**H7(c)**	PPI * IL → AITL	−0.096 **	IL dampens the positive relationship between PPI and AITL
**H8(a)**	FNPO * ADT → AITL	0.230 ***	ADT strengthen the positive relationship between FNPO and AITL
**H8(b)**	PSD * ADT → AITL	0.268 ***	ADT strengthen the positive relationship between PSD and AITL
**H8(c)**	PSS * ADT → AITL	0.236 ***	ADT strengthen the positive relationship between PSS and AITL

*** *p* < 0.001. ** *p* < 0.010. * *p* < 0.050.
